# Men's experiences of receiving a prostate cancer diagnosis after opportunistic screening—A qualitative descriptive secondary analysis

**DOI:** 10.1111/hex.13567

**Published:** 2022-07-27

**Authors:** Linda Gellerstedt, Ann Langius‐Eklöf, Nazmije Kelmendi, Kay Sundberg, Åsa G. Craftman

**Affiliations:** ^1^ Department of Neurobiology, Care Sciences and Society Division of Nursing, Karolinska Institutet Stockholm Sweden; ^2^ Stockholm County Council, Academic Primary Health Care Center Stockholm Sweden; ^3^ Department of Nursing Science Sophiahemmet University Stockholm Sweden

**Keywords:** experiences, opportunistic screening, prostate cancer, qualitative research, secondary analysis

## Abstract

**Background:**

Prostate cancer is one of the most common types of cancer in men and could occur without symptoms. Screening has been debated but remains controversial and, in most countries, organized population‐based screening does not exist. The aim of this study was to describe men's experiences of receiving a prostate cancer diagnosis after opportunistic screening.

**Methods:**

This study is a secondary analysis from interviews with 17 men (aged 56–80 years) who had undergone curative treatment for prostate cancer. Data were collected in an urban region of Sweden through interviews conducted face to face or by telephone. An inductive content analysis was used with Consolidated criteria for Reporting Qualitative research as a reporting checklist.

**Results:**

Two main categories were identified. *Screening is a lifesaver enclosed by ethical dilemmas* reflects how men considered screening as a lifesaving test. Testing was surrounded by injustice and an eagerness to encourage other men to undergo screening. *Facing challenges during diagnosis* reflects the men's experiences of being in an unknown field yet expected to engage in decision‐making concerning appropriate treatment. Receiving the diagnosis rendered mixed emotions about having a cancer disease, that the treatment could cause lifelong symptom distress and the men described being hesitant to talk about their diagnosis.

**Conclusions:**

The findings highlight men's opinions about screening and that the lack of routine screening represents injustice. The men considered this as an ethical question of lifesaving justice, while stakeholders may argue that screening could lead to unnecessary suffering and overtreatment. Men do not always talk openly about their diagnosis, linked to the fact that it concerns intimate areas. It is important to balance the information in relation to shared decision‐making regarding treatment. Health care professionals have an ethical responsibility to support and coach the patient in their decision.

**Patient or Public Contribution:**

This study was based on interviews with men who had experienced a diagnosis of prostate cancer.

## 1 INTRODUCTION

Prostate cancer is one of the most common types of cancer in men in Western countries,^1^ and the second most common cancer in men worldwide, accounting for an estimated 1.3 million new cases diagnosed in 2018.^2^ Prostate cancer has a long period of latency, up to 15–20 years, during which the disease is histologically present but has not yet become symptomatic.^3^ Therefore, it is often referred to as the silent man's disease.^4^ In Sweden, as in most western countries, an organized population‐based screening programme where the men are invited for prostate‐specific antigen measurement (PSA) does not exist, but men choose frequently to undergo opportunistic screening. Opportunistic screening means that the men are offered a PSA test during a visit to a physician or that men request a PSA test. Therefore, the decision to take the test potentially depends on the individual's own knowledge.^5^, ^6^ Screening is extensively debated and remains controversial.^7^ While screening for prostate cancer may reduce the risk of mortality, it is associated with side effects involving false‐positive results, biopsy complications and overdiagnosis.^8^, ^9^, ^10^ The overdiagnosis, not to be confused with false‐positive results, refers to when the prostate cancer never progresses or progresses slowly and renders the possibility that men die of other causes before it becomes symptomatic.^6^, ^8^, ^11^

During the last decade, advances have been made regarding curative treatment of prostate cancer.^12^ The available treatment options today related to cancer stage are watchful waiting (active surveillance), hormone therapy, radiation and surgery. Regardless of treatment choice, it may lead to distressing symptoms such as erectile dysfunction and urinary incontinence.^8^, ^13^ Receiving a cancer diagnosis may generate emotional reactions that make reading and absorbing information about treatment difficult.^12^ Thus, patients' preferences regarding cancer treatment decisions need to be considered. The concept of shared decision‐making comprises a model for decisions about treatment in medical encounters between the patient and the physician.^14^, ^15^ Based on the fact that there are various treatment options for prostate cancer, with different side effects, it is customary to include patients in a shared decision‐making process.^16^, ^17^

During the analysis of data in an interview study with men treated for prostate cancer aiming to identify symptoms and self‐care during the first year of survivorship (unpublished), the authors of the present study identified other important areas from a research ethical perspective. The men expressed extensive narratives concerning their emotional distress influencing the period after screening and receiving the diagnosis. In a review that describes men's motives and doubts, facing the decision to undergo opportunistic screening indicates that men were eager to detect signs of cancer in time, even when they knew the drawbacks of the test, such as false‐positive results.^18^ However, men's experiences after opportunistic screening are scarcely described.^19^, ^20^ Furthermore, men's decision‐making ability to evaluate treatment options may be affected by emotional reactions. In encounters with men regarding shared decision‐making, health care professionals should make sure that the patient has understood all the information before a shared decision.^17^

## 2 AIM OF THE STUDY

This study aimed to describe men's experiences of receiving a prostate cancer diagnosis after opportunistic screening.

## 3 METHODS

### 3.1 Design

The study used a descriptive design applying a qualitative secondary analysis of transcripts from individual interviews. Secondary analysis is suggested when data from a primary study comprise elaborations deviating from the original study aim to fully report and utilize the potential of existing data.^21^, ^22^ This study followed the Enhancing Transparency of Reporting the Synthesis of Qualitative research framework^23^ and the Consolidated criteria for Reporting Qualitative research checklist.^24^

### 3.2 Participants and setting

The manager at one urban university hospital in Stockholm, Sweden, serving both rural and urban parts of the region with specialist care, was provided information about the study, and permission to recruit participants was obtained. Inclusion criteria were men diagnosed with prostate cancer who had completed a curative treatment during the past year and were considered cognitively able to participate in an interview and could speak and understand the Swedish language. An invitation letter about the study was shared with 25 patients by two nurses at the clinic and through a group meeting with potential participants who fulfilled the inclusion criteria. Those 14 who accepted the invitation sent their response in a prestamped envelope and were thereafter contacted by a researcher. At the postsurgery group meeting, the participating seven patients were informed about the study and three agreed to participate. In total, 17 men who fulfilled the inclusion criteria accepted the invitation; see Table 1 for the demographics of the participants.

**Table 1:** Characteristics of participating men

*N* = 17	*n*
Age in years (range)	56–80
Marital status	
Partner	16
Single	1
Employment	
Employed	3
Retired	14
Education level	
Primary school	1
College	8
University	8
End of treatment in months at the interview	
<3	6
<6	6
<9	1
≤12	3
15	1

### 3.3 Data collection

The interview guide used in the primary study was developed within the research group and was tested in two pilot interviews. All interviews were conducted between October 2019 and June 2020 by a female researcher and district nurse with extended experience of health coaching sessions in primary health care. Time and place were chosen by the participant. In total, 17 men were interviewed. Eleven men participated in face‐to‐face interviews: two in their workplaces, three in their homes and six in the researcher's office. The remaining six interviews were performed as telephone interviews due to the Covid 19 restrictions. Each interview started with the question ‘Could you please tell me how you have experienced the time after your treatment and how do you feel now?’ In answering this question, the participants richly described their experience about the screening process and receiving a diagnosis. The men gave rich narratives concerning the period of screening and diagnosis, which yielded a new aim for this secondary analysis. The interviews lasted between 29 and 78 min (mean of 47 min) and were audio‐recorded with the patients' approval. They were thereafter transcribed verbatim in close connection to the interviews.

### 3.4 Data analysis

The transcribed interview text was analysed by two of the authors using inductive content analysis described by Elo and Kyngäs.^25^ First, the researchers individually read the transcribed interviews several times to become familiar with and gain a thorough understanding of the content. In the next phase, the researchers together, side by side, read the text and identified and marked units of analysis, consisting of sentences or paragraphs in the text that were related to the aim. Thereafter, an open coding process was performed. This process involved labelling the content of the units of analysis and codes were made in the margins of the document. After the open coding, all codes were checked against the text and through a constant comparison of similarities and differences between codes. Thereafter, the codes were sorted and grouped together into five formulated subcategories. An abstraction process was conducted by flexibility, meaning moving back and forth between transcribed data, units of analysis, codes and subcategories^26^ that yielded two categories. Finally, categories and subcategories were discussed within the research team until consensus was achieved on both interpretations and abstractions of data.

### 3.5 Rigour

Transferability was strengthened by presenting the context, the demographics of the participants (Table 1) and the purposive sampling. Credibility was supported by systematically following Elo and Kyngäs^25^ descriptions of analysis, by clearly presenting the analysis process, both in the text and in Tables 2 and 3, and the discussions within the research team regarding consensus in the analysis. Each step of the analysis process was characterized by flexibility and repetitive verification against the transcribed interviews, codes and categories, which enhanced reliability and dependability. To increase confirmability, quotations were used to illustrate the findings.

**Table 2:** Examples from the analysis process

Analyse units for open coding	Subcategories	Main categories
‘The diagnosis came out of the blue. I had no symptoms and suddenly I was a “hospital object”, I became a patient …’ (P 11).	The test gave voice to the disease	Screening is a lifesaver enclosed by ethical dilemmas
‘It is cancer we are talking about so as to let a number of thousands of men pass away in a rather disgusting way as my father did, I think is unforgivable especially as it is so easy to screen’ (P 5).	An ethical conflict—men's opinions of screening	
‘There's an end to this too, the doctor said we'll fix this, and you'll get ten years to live something like that. I have never thought in those paths before, that life ends, it does, you do not think about it until you get a disease…’ (P 12).	Existential thoughts about circumstances of life	Facing challenges during diagnosis
‘I cannot assess my own situation before treatment, there are others who know more about this. It is like when you fly, somehow you assume and hope the pilot knows the job’ (P 4).	Being a part of the treatment decision	
‘It is probably the case that you probably do not talk about this in the same way, there you are probably old‐fashioned as I said, I mean there are probably many who cannot talk to anyone about this at all. It is not directly like talk about a common cold’ (P 14).	Talking about an intimate diagnosis	

**Table 3:** Presentation of findings

Main categories	Subcategories
Screening is a lifesaver enclosed by ethical dilemmas	– The test gave voice to the disease – An ethical conflict—men's opinions of screening
Facing challenges during diagnosis	– Existential thoughts about circumstances of life – Being a part of the treatment decision – Talking about an intimate diagnosis

### 3.6 Ethical considerations

Ethical approval was obtained for the study from the Ethical Review Authority (Dnr: 2019‐00379), and the research followed the Declaration of Helsinki.^27^ Before inclusion in the study, all participants were informed, both verbally and in written form, about the aim: to describe the patients' experiences during the first year after curative treatment. The voluntary nature and confidentiality of participation and their right to withdraw at any moment were also emphasized. They were also made aware that confidentiality would be preserved and that the quotes from the interviews would be formulated to protect the identity of the participants. All men signed the informed consent form before the interview started. The informed consent aligns with the objectives of the secondary analysis.

## 4 RESULTS

The findings are presented in two main categories and five underlying subcategories presented in Table 3.

### 4.1 Screening is a lifesaver enclosed by ethical dilemmas

#### 4.1.1 The test gave voice to the disease

The men stated the desire for early detection of a possible cancer as one major motive to undergo screening despite being in good health generally. Some of the men had arranged the screening by themselves, encouraged by friends and relatives, but in other cases, men had been invited by the occupational health care. Some men voiced how other men had encouraged them to take the test based on the fact that one could have the disease without any symptoms.

Regardless of the motive for taking the test, or if it was on an annual basis, several of the men in this study described a feeling of unpreparedness when the test result was cancer. According to the men's descriptions, the diagnosis was perceived as coming out of the blue and it was a challenge to suddenly become a patient from 1 day to the next. As a patient, the men expressed the adaptation to the health care system, as a feeling of being objectified which overwhelmed them:

The diagnosis came out of the blue. I had no symptoms and suddenly I was a ‘hospital object’, I became a patient …. (P 11)

#### 4.1.2 An ethical conflict—men's opinions of screening

Several of the men described the opportunistic screening in terms of injustice and equality compared to the Swedish national programme of mammography. The men stated that there is a need for a national information campaign to highlight the need for men to undergo screening. They emphasized that screening for prostate cancer should be routinely offered to everyone as an organized population‐based screening programme:

I think that screening should be introduced, from a socioeconomic point of view, because it is insane to let people who would otherwise be fully healthy burden the health care system, handling the prostate cancer at an early stage can be so much easier and faster than all years of treatments as one will need otherwise. (P 5)

Some participants described the complexity surrounding screening, such as the fact that a high PSA can be caused by things other than prostate cancer, and that this posed a risk for overdiagnosis. However, those arguments were described as subordinate to the possibility of detection of the disease in time.

### 4.2 Facing challenges during diagnosis

#### 4.2.1 Existential thoughts about circumstances of life

When being diagnosed, the men described how they faced different challenges. They described existential thoughts from a survival perspective about having a potentially lethal disease, whether the cancer was detected in time and the possibility that treatment could cause lifelong symptom distress. Although all men in this study had undergone curative treatment, they expressed how the diagnosis had raised existential thoughts about life as they know it, death and one's own mortality in a novel way. One man described this as follows:

there's an end to this too, the doctor said something like we will fix this, and you will get ten years to live. I have never thought in those paths before, that life ends, it does, you do not think about it until you get a disease…. (P 12)

When viewing the information about treatment options, surviving was described as the main priority and thus most important; the possible symptom distress was something the men expressed that they could adjust to. The context of sexuality was described as an essential and joyful area of life; however, if this was no longer an option after treatment, it was seen as a low price to pay in exchange for life:

… I did rank it in a kind of order, to survive is number one, continence is number two and potency got to be number three. (P 2)

#### 4.2.2 Being a part of the treatment decision

The men described receiving a lot of information about different treatment options. Regarding the decision, men described themselves as being complete novices in an unknown field and how this was causing doubts regarding their capacity to value appropriate treatment options. Men stated that their preunderstanding of treatment options was affected by narratives from friends and relatives who had undergone treatment for prostate cancer. Yet, some men stated that they knew that treatment had improved over the years. Several men described the shared decision as twofold: they appreciated that they had the opportunity to be a part of the decision of treatment; however, the decision was difficult to make. One man described this as follows:

I was given three options to decide on, to wait, to have surgery or to undergo radiation treatment. They informed me but I thought it was hard for me to make the decision. (P 12)

Thoughts of adverse outcomes related to different options affected their decision, and some men felt disregarded in the decision on the basis that the doctor's appointment was too short and perceived as stressful. Some men expressed a need to further discuss and reflect on the options and symptom distress before the decision.

#### 4.2.3 Talking about an intimate diagnosis

Some of the men were reluctant and hesitated to talk openly about their diagnosis. The cause of this was described by the men as related to the intimacy of the diagnosis, as it was associated with, for instance, loss of sexual function and the risk of incontinence. For example, some men expressed how they adjusted their wording about the disease when women were present, and others stated that they only talked about it with the closest family. This was exemplified as follows:

I think before I tell anyone about my prostate cancer, it is not the Achilles tendon directly … I do hesitate to talk about my diagnosis and treatment. (P 1)

On the other hand, some men described the importance of talking openly to others about their prostate cancer diagnosis to raise awareness of the disease and the importance of being tested. Furthermore, they had experienced that opening up and talking about it had made them aware that there were many others in their vicinity who had experiences of prostate cancer in the present or in the past.

## 5 DISCUSSION

This study aimed to describe men's experiences of receiving a prostate cancer diagnosis after opportunistic screening. The findings reveal strong opinions about opportunistic screening and that every man has the right to be screened for prostate cancer. The lack of organized population‐based screening was considered a poor political decision when life was at stake and was expressed as an injustice compared to mammography. As in our study, Kirkegaard et al.^20^ found that men were not concerned about the risk of overtreatment and instead focused on the perceived benefits of early detection. These perspectives illustrate a conflict between men's opinions of screening and public health recommendations. The narrative in the findings implies a gap in the general information about PSA testing including potential adverse effects such as the risk of infections and eventual false‐positive results. The Swedish National Board of Health and Welfare^6^ finds that opportunistic screening in men is increasing in Sweden, and this may be explained to some extent by the fact that men are eager to get screened.

In line with previous research, our findings show how an unexpected, life‐threatening diagnosis without recognized symptoms raises existential emotions.^11^, ^18^, ^28^ Having prostate cancer can represent a form of vulnerability due to its connotations of intimacy, including erectile dysfunction and incontinence. Accordingly, men in this study voiced that they hesitated to talk about the diagnosis with people other than their closest relatives. When seeking health care support, men's inherent masculinity norms and gender roles are identified as considerable obstacles.^29^, ^30^

Patients' experiences of illness are unique, and they are the ones who will benefit from or suffer the consequences of the medical treatment^31^ and, therefore, shared decision‐making is stipulated by law for health care^32^ and is recommended by WHO.^33^ However, our findings show that shared decision‐making is complex. The men felt uncertain of whether they had enough information and knowledge as laymen, and in some cases, men expressed being doubtful of their decision‐making capacity. The process encompasses unavoidable asymmetry as physicians know more about treatment than patients.^31^ It is important to recognize the complexity of patients' abilities and preferences for shared decision‐making. Patients who clearly renounce shared decision‐making must be respected, and it is crucial to not perceive this as an additional burden.^34^ Therefore, health care professionals should be supportive and devote sufficient time for patient encounters. Furthermore, by keeping a person‐centred perspective in mind, it is essential that health care professionals assist men regardless of whether it concerns decisions about screening or treatment. Health care professionals must also be careful not to insist that they know patients' needs better than the patients themselves. The results from this qualitative study may not be transferable to other settings; yet, these men's experiences provide important perspectives when developing and evaluating supportive care interventions.

All interviews were planned to be conducted face to face, but due to the Covid‐19 pandemic, six interviews were conducted by telephone. It is possible that the changed modality affected interview dynamics and outcomes. However, the men felt and some even stated feeling comfortable in describing and sharing their situation during the interviews with the female researcher. The inclusion criterion of the Swedish language may entail a limitation, since non‐Swedish‐speaking persons were excluded. The method of secondary analysis is described as a valuable addition to conducting a primary study and/or exploration of a linked perspective, but has some shortcomings.^21^ For example, no questions can be added to the question guide, and no follow‐up questions can be asked to develop specified answers since the data have already been collected. However, out of respect for the participants' views and responses, we considered it important to explore these rich data through the lens of a new aim.

## 6 CONCLUSIONS

The findings highlight men's opinions about screening and that the lack of routine screening represents injustice. The men considered this as an ethical question of lifesaving justice, while stakeholders may argue that screening could lead to unnecessary suffering and overtreatment. Men do not always talk openly about their diagnosis, linked to the fact that it concerns intimate areas. It is important to balance the information in relation to shared decision‐making regarding treatment. Health care professionals have an ethical responsibility to support and coach the patient in their decision.

## AUTHOR CONTRIBUTIONS

Ann Langius‐Eklöf and Kay Sundberg designed the study, Nazmije Kelmendi collected data, Linda Gellerstedt and Åsa G. Craftman performed the analysis and the analysis was checked by Ann Langius‐Eklöf, Kay Sundberg and Nazmije Kelmendi. All authors contributed to the writing of the manuscript, and all authors read and accepted the last version of the manuscript.

## CONFLICT OF INTEREST

The authors declare no conflict of interest.

## ETHICS STATEMENT

Ethical approval was obtained for the study from the Ethical Review Authority (Dnr: 2019‐00379).

## Data Availability

The data that support the findings of this study are available from the corresponding author, (Åsa G. Craftman), upon reasonable request.
